# Trends in the quality of antenatal care in India: Patterns of change across 36 states and union territories, 1999–2021

**DOI:** 10.7189/jogh.14.04188

**Published:** 2024-10-18

**Authors:** Hwa-Young Lee, Akhil Kumar, Anoop Jain, Rockli Kim, S V Subramanian

**Affiliations:** 1 Graduate School of Public Health and Healthcare Management, The Catholic University of Korea, Seoul, South Korea; 2 Catholic Institute for Public Health and Healthcare Management, The Catholic University of Korea, Seoul, South Korea; 3 Faculty of Arts and Sciences, University of Toronto, Toronto, Ontario, Canada; 4 Department of Environmental Health, Boston University School of Public Health, Boston, Massachusetts, USA; 5 Division of Health Policy and Management, College of Health Science, Korea University, Seoul, South Korea; 6 Interdisciplinary Program in Precision Public Health, Department of Public Health Sciences, Graduate School of Korea University, Seoul, South Korea; 7 Harvard Center for Population and Development Studies, Cambridge, Massachusetts, USA; 8 Department of Social and Behavioral Sciences, Harvard T. H. Chan School of Public Health, Boston, Massachusetts, USA

## Abstract

**Background:**

Antenatal care (ANC) quality is important to maternal and neonatal mortality. However, trends in the quality of ANC received by pregnant women in India have been understudied. This paper seeks to fill this gap by examining the long-term patterns nationwide and the state-specific prevalence of inadequate ANC quality received by pregnant women in India.

**Methods:**

We utilised data from four National Family Health Surveys (NFHS) conducted in 1999 (NFHS-2), 2006 (NFHS-3), 2016 (NFHS-4), and 2021 (NFHS-5) across India’s 36 states/union territories (UTs). The sample includes mothers who had given birth within three years (NFHS-2) and five years (NFHS-3, NHFS-4, and NFHS-5) before each survey. We define inadequate ANC quality as not completing seven essential ANC services (weight measurement, blood pressure measurement, urine sampling, blood sampling, provision of iron supplements, provision of tetanus vaccination, and ultrasound scans) during pregnancy. We calculated the standardised absolute change to quantify the change in the share of women receiving inadequate quality ANC nationally and by each state/UT. Additionally, we estimated the population headcount of mothers who received inadequate-quality ANC in 2021 and identified the socioeconomic correlates associated with inadequate ANC quality.

**Results:**

The prevalence of inadequate ANC quality substantially declined between 1999–2021, from 84.8% (95% confidence interval (CI) = 84.1–85.5) to 28.8% (95% CI = 28.5–29.2). However, between-state inequality in ANC quality has increased over this time. We identified a weak correlation between prevalence and population headcounts in 2021. Socioeconomically disadvantaged groups exhibited a higher prevalence of inadequate quality of ANC than less disadvantaged groups.

**Conclusions:**

The proportion of pregnant women receiving inadequate ANC quality has decreased over time throughout India. However, multi-faceted efforts at national and state levels are necessary to enhance the effectiveness of existing policies. Additionally, innovative and targeted approaches are required to ensure the timely and equitable provision of high-quality ANC.

Reducing maternal and neonatal mortality is one of the major targets of the Sustainable Development Goals (SDGs). Antenatal Care (ANC) is a critical predictor of maternal and neonatal mortality as it can help detect high-risk pregnancies, which can then be referred to higher-level health care facilities, a step that is key in averting maternal and neonatal deaths [1,2]. Increased ANC utilisation worldwide could help explain global reductions in maternal and neonatal mortality since 2000 [3]. However, the decrease in maternal and neonatal mortality has not been proportional to the increased uptake of service utilisation [4,5], which prompted a shift in policy discussion away from the mere expansion of service coverage and towards improvement in the quality of services [6].

There is an interdependent relationship between service utilisation and quality. Evidence suggests that when patients perceive health care services as substandard, they lose trust, discouraging active utilisation of care due to fear of poor outcomes [7]. Conversely, low utilisation of health services diminishes opportunities for on-site learning and reduces health care workers’ motivation, further deteriorating service quality [8]. Recognising the importance of health service quality, SDG 3.8 explicitly emphasises universal health coverage for high-quality health care services as an integral component. The World Health Organization (WHO) and World Bank have advocated for enhancing ‘effective coverage,’ a metric that combines the need for, utilisation of, and quality of services rather than ‘crude coverage,’ which focuses solely on access to services [9–12].

Approximately 17.5% of global maternal and neonatal deaths and stillbirths in 2020 occurred in India [13]. While India is on track to achieve the SDG target for reducing maternal mortality rate by 2030 [14,15], it might not attain the SDG target for reducing neonatal mortality rates at the current rate of reduction [16]. India initiated efforts to improve ANC service quality through the nationwide Child Survival and Safe Motherhood program introduced in 1992–93. However, much of these efforts were still dedicated to enhancing ANC utilisation (Table S1 in the **Online Supplementary Document**) [17,18]. The National Rural Health Mission addresses ANC utilisation and quality in rural areas [19,20]. Initiatives like the Prime Minister’s Overarching Scheme for Holistic Nutrition Abhiyaan and Anaemia Mukt Bharat focus on ensuring iron and folic acid supplementation during pregnancy [21,22]. In addition to these nationwide efforts in India, many states have developed policy initiatives, such as Odisha’s mobile health unit, that aim to improve the quality of health care services for their respective populations (Table S1 in the **Online Supplementary Document**) [23,24].

While numerous studies have investigated trends in utilising maternal services, such as the completion of specific numbers of ANC visits or delivery at health facilities in India [25–28], knowledge gaps remain concerning the quality of care provided. Only a few studies have examined the prevalence of specific ANC services received by pregnant mothers, and these studies relied on limited sample sizes derived from specific geographic areas and short timeframes [12,29–31]. Furthermore, given the substantial variations in development indicators, including health, across different states/union territories (UTs), it is plausible that the quality of maternal service and their progress also vary. However, the state-specific long-term changes in the quality of ANC services have yet to be thoroughly examined. In India, the states are the primary political units at which federal policies are implemented and the budgetary allocations for different development sectors are decided. Therefore, assessing the nationwide and sub-national progress in the quality of ANC services will enable India to adjust its plans to meet SDG target three of achieving universal health coverage of quality care.

We conducted this study to address three primary research questions. First, what are the long-term trends in the prevalence of pregnant women who have not received the full package of seven ANC services (henceforth referred to as ‘inadequate quality of ANC’) across India and each of its 36 states and UTs and what are the disparities in these trends among 36 states/UTs disparities between 1999–2021. Second, what is the actual population headcount of women who received inadequate quality ANC for all of India and each of the states/UTs? The final research question is: how does the prevalence of inadequate quality of ANC in 2021 vary across basic demographic and socioeconomic characteristics?

## METHODS

### Study design and participants

This cross-sectional analysis used data from the National Family Health Surveys (NFHS) at four different time points: 1999 (NFHS-2), 2006 (NFHS-3), 2016 (NFHS-4), and 2021 (NFHS-5) [18,32–34]. NFHS data are publicly accessible and were obtained from the Demographic and Health Survey website. The Demographic and Health Survey provides maternal and child health information collected through interviews of women aged 15–49 years. Each wave of NFHS is nationally representative, covering all states and UTs in India using a multistage stratified cluster sampling design. In the first stage, the primary sampling units (or clusters) corresponding to villages in rural areas and census enumeration blocks in urban areas were selected by probability proportional to cluster size [35]. This was followed by random household selection from the household list using systematic sampling with equal probability. More details on the survey design are described in previous work [35].

The study sample comprised mothers who had given birth within three years preceding the survey in NFHS-2 and within five years preceding the survey in NFHS-3, NFHS-4, and NFHS-5. We restricted the study sample to the mother’s most recent birth to mitigate recall bias regarding the specific ANC items received during their pregnancy. Additionally, we included only those mothers who had received ANC at formal health facilities to monitor the health system’s quality. NFHS data collection was approved by the International Institute for Population Studies Institutional Review Board. This study did not constitute human subject research as per the Harvard Longwood Campus Institutional Review Board and was thus exempt from a full review.

### Outcome

Our primary outcome of interest was ANC quality. We focused our assessment on technical quality, typically assessed through measures such as adherence to clinical guidelines or application of evidence-based practices [5]. We constructed an ANC quality indicator based on the specific ANC services that impact maternal and child mortality using the following steps. First, we reviewed WHO guidelines for the essential ANC services that are crucial to the detection of pregnancy risks, including hypertension, pre-eclampsia, infection, anaemia, and nutritional deficiencies [36]. We then identified the corresponding ANC services captured in the NFHS, resulting in seven items: weight measurement, blood pressure reading, urine sampling, blood sampling, iron tablets/syrup supplementation, tetanus injection, and ultrasound test. These seven service items, while not exhaustive, are associated with reduced neonatal mortality and have been used as ANC quality indicators in other studies [37,38]. Finally, we defined inadequate ANC quality as a binary indicator denoting whether the full package of seven ANC items was received. We use the term ‘adequate quality’ rather than ‘good quality’ because the information on ANC services available in the NFHS data does not exhaustively cover all the services recommended by WHO.

### Constructing comparable state estimates

India’s subnational boundaries have evolved over the past three decades. In 1999, there were 25 states and seven UTs, which expanded to 28 states and eight UTs in 2021, making state/UTs comparisons over time complicated. To address this issue, we assigned districts surveyed in NFHS-2 and NFHS-4 to their current state/UTs as they existed in NFHS-5 (2021). For NFHS-3, pre-existing state/UT values were applied to the new states/UTs due to the unavailability of district information. This methodology enables older state geometries to reflect current estimates as accurately as possible, allowing meaningful policy insights at the state level. This methodology has been verified in previously published work [35].

### Analysis

We calculated the weighted percentage and 95% confidence interval (CI) of mothers who have not completed all seven ANC items, both nationally and at the state level, using the following equation:

Prevalence of inadequate ANC quality = (mothers not completing seven ANC items during pregnancy/mothers receiving ANC in formal health facilities) × 100.

The same calculation was also performed for each of the seven individual ANC items. Each wave of the NFHS includes a weight factor for the multi-stage stratified cluster sampling design. We multiplied each respondent’s data by the corresponding weight to ensure that the sample data accurately represents the Indian population. This approach corrects for over- or under-sampling of specific groups, thereby enhancing the validity and generalizability of our findings. To quantify the change in the prevalence of inadequate quality of ANC over the course of four time periods, absolute change (AC) (in percentage points) and standardised absolute change (SAC) were calculated:

*AC = P_t_ − P_t − n_*

*SAC = (P_t_ − P_t − n_)/N*

*P_t_* denotes the prevalence in year *t*, while *P_t_*_ −_*_ n_* denotes the prevalence in *n* years prior to the reference year *t*. N represents the number of years between any two surveys. A negative AC or SAC indicates improved ANC quality, while a positive AC or SAC indicates a worsening trend in providing adequate quality of ANC. We descriptively assessed the correlation between the magnitude of SAC in the prevalence during the study years (between 1999–2021) and the prevalence at the baseline of study years (1999) using scatter plots.

We used boxplots and the corresponding interquartile range (IQR) to show the between-state inequality in the prevalence of inadequate quality of ANC. We examined the extent to which state/UT ranking in terms of inadequate ANC quality is correlated across various years using correlation tests and by creating a graph of the change in the ranking of individual states in the prevalence of inadequate quality of ANC across the study years.

The population headcount of mothers who received inadequate quality of ANC services was estimated at the all-India level and for each state/UT in 2021. This estimation was based on the combination of the microdata and Census of India Population projections. We used the total population of women aged 15–49 years for 2021, replicating the integrated public use microdata series methodology with an assumed total population of 362 865 992 women in this age group across India [39]. This approach has been validated in previously published work [35]. We conducted a visual and quantitative assessment to explore whether states/UTs with a higher prevalence of inadequate ANC correlate with a higher absolute headcount of mothers who have received inadequate ANC.

Finally, we examined the socioeconomic correlates of inadequate ANC quality. These include multiple births, the sex of the baby, birth order, the mother’s pregnancy age, educational attainment, household wealth level, social caste, marital status, and place of residence. All analyses were performed using Stata, version 17.0 (StataCorp, College Station, Texas, USA) [40].

## RESULTS

### Sample characteristics

After excluding ANC care at home, the number of mothers who received ANC for their last birth at formal health facilities was 15 061, 23 137, 127 515, and 131 842 in 1999, 2006, 2016, and 2021, respectively. We did not exclude any observations, as none were missing on all seven items. As a result, the final analytic sample size remained the same as the original sample size (Table S2 in the **Online Supplementary Document**).

The characteristics of mothers who sought ANC services at formal health facilities changed over the study period. The proportion of pregnant mothers before age 20 declined from 23.6% in 1999 to 10.0% in 2021. Overall, mothers’ education level improved, with the proportion of those with no formal education decreasing from 37.9% to 18.7% and those with secondary education increasing from 38.2% to 51.4%. The proportion of mothers from households with the lowest and the second-lowest wealth quintile and from scheduled tribes and other backwards castes increased. The percentage of mothers living in rural areas also increased (Table S2 in the **Online Supplementary Document**).

### Patterns of change in the prevalence of mothers who received inadequate quality of ANC

**Figure 1:**
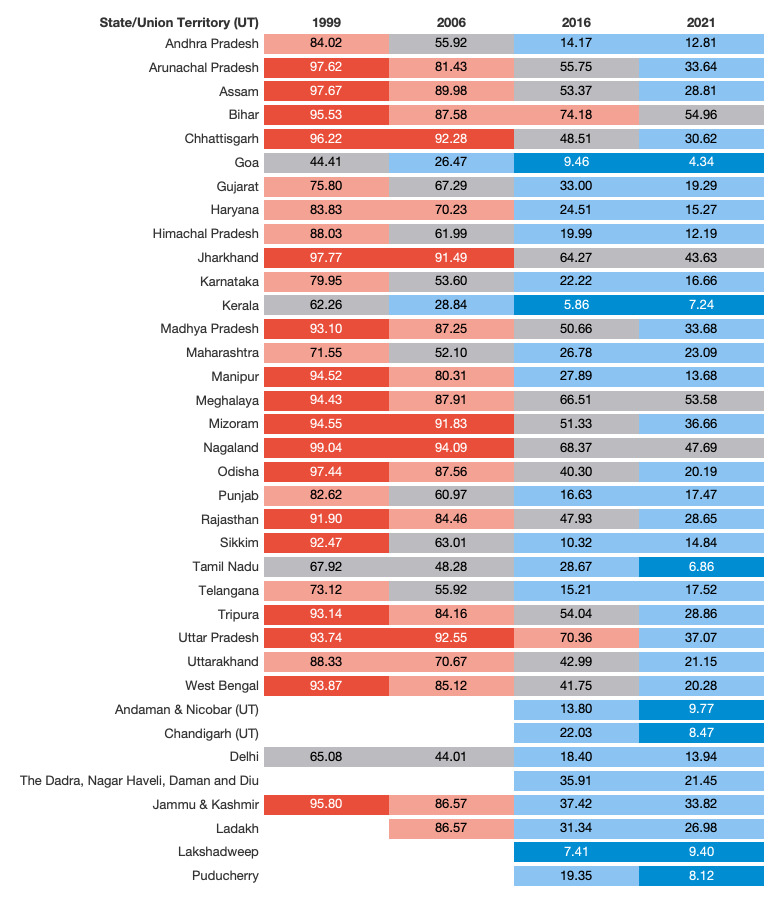
Inadequate antenatal care prevalence for states and union territories of India, 1999–2021.

**Figure 2:**
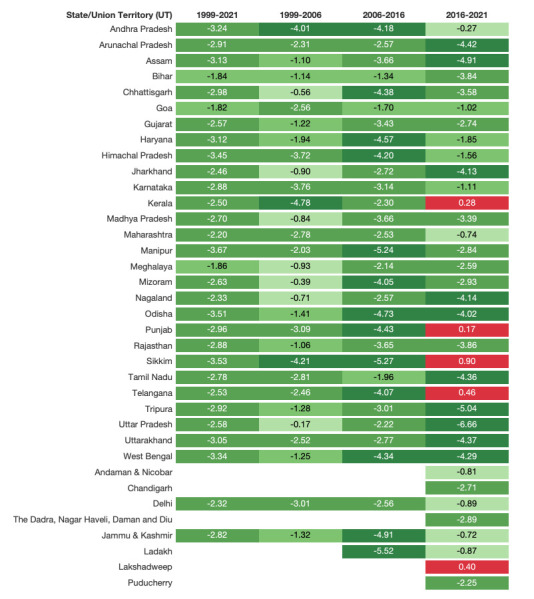
Standardised absolute change for inadequate antenatal care prevalence for states and union territories of India between 1999–2006, 2006–16, 2016–21, and 1999–2021.

The overall prevalence of mothers who did not receive the full package of ANC services fell from 84.8% (95% CI = 84.1–85.5) in 1999 to 28.8% (95% CI = 28.5–29.2) in 2021 (Table S3 in the **Online Supplementary Document**). In 1999, the prevalence of mothers who did not receive all seven ANC service items was above 90.0% in over half of the 30 states (**Figure 1**, Table S3 in the **Online Supplementary Document**). However, this was only the case in five states by 2006 (Chhattisgarh, Jharkhand, Mizoram, Nagaland, and Uttar Pradesh). By 2016, no state or UT had a prevalence of mothers not receiving all seven ANC items above 90.0%. Among the 17 states that showed a prevalence of over 90.0% in 1999, six states (Andhra Pradesh, Himachal Pradesh, Manipur, Odisha, Sikkim, and West Bengal) achieved a decline of 70.0 percentage points or more between 1999–2021, of which Manipur showed the largest decline (AC = –80.8, SAC = –3.6) (**Figure 2**, Table S4 in the **Online Supplementary Document**). On the other hand, Bihar and Meghalaya only achieved a decline of slightly higher than 40.0 percentage points (SAC = –1.8 and –1.8, respectively), resulting in a prevalence of inadequate quality of ANC of approximately 53.0% in 2021. In most states, the most significant progress occurred between 2006–16, with 13 achieving AC of more than 40.4 percentage points or SAC of more than 3.0 percentage points (**Figure 2**, Table S4 in the **Online Supplementary Document**).

Among the seven ANC items, ultrasound was the least performed service in 1999, with 78.9% (95% CI = 78.1–79.7) of mothers not receiving it. However, by 2021, the prevalence gap between ultrasound provision and the other six ANC services had narrowed (Tables S5–11 in the **Online Supplementary Document**). The progress in the provision of individual ANC services varied across different states. For example, the percentage of women not weighed during ANC visits decreased to below 5.0% in 2021, except for Bihar (Table S5 in the **Online Supplementary Document**). On the other hand, progress in iron supplementation varied across states: Manipur experienced a substantial decrease in the percentage of women not receiving iron supplements, from 38.7% to 4.1% during the study period, while Bihar experienced a relatively minimal decrease from 36.4% to 22.3% over the same period (Table S10 in the **Online Supplementary Document**).

**Figure 3:**
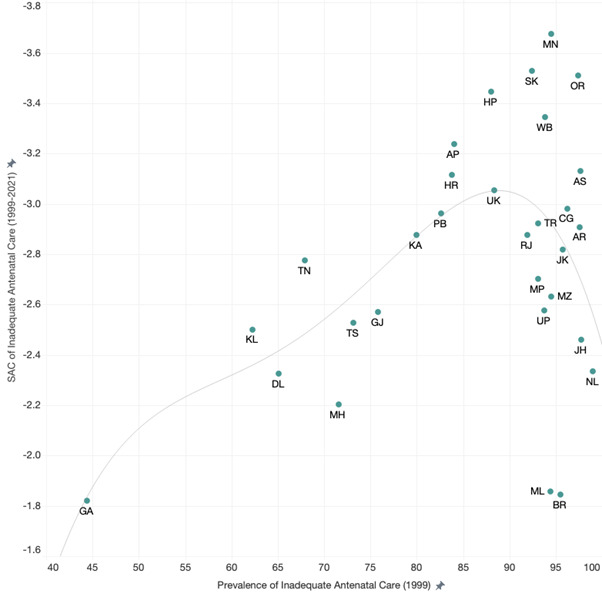
Relationship between 1999 prevalence of inadequate antenatal care and standardised absolute change of inadequate antenatal care for 1999–2021. AN – Andaman and Nicobar, AP – Andhra Pradesh, AR – Arunachal Pradesh, AS – Assam, BR – Bihar, CH – Chandigarh, CG – Chhattisgarh, DH – Dadra and Nagar Haveli and Daman and Diu, GA – Goa, GJ – Gujarat, HR – Haryana, HP – Himachal Pradesh, JK – Jammu and Kashmir, JH – Jharkhand, KA – Karnataka, KL – Kerala, LK – Ladakh, LD – Lakshadweep, MP – Madhya Pradesh, MH – Maharashtra, MN – Manipur, ML – Meghalaya, MZ – Mizoram, DL – NCT Delhi, NL – Nagaland, OR – Odisha, PY – Puducherry, PB – Punjab, RJ – Rajasthan, S – Sikkim, TN – Tamil Nadu, TL – Telangana, TR – Tripura, UP – Uttar Pradesh, UK – Uttarakhand, WB – West Bengal.

**Figure 4:**
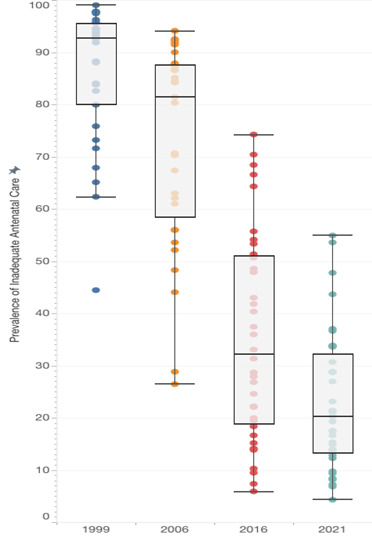
Summary distribution of inadequate antenatal care prevalence across states/union territories of India, 1999–2021. The median is represented by a dark line inside each rectangle. The interquartile range (IQR) is shown by the length of the rectangle. The extent of the whiskers shows data that is 1.5 times the IQR. Outliers are shown outside the extent of the whiskers when they are 1.5 times greater or smaller than the IQR.

We found that SAC between 1999–2021 was positively correlated with the initial year prevalence among states where the 1999 prevalence was below 90.0%. In contrast, an inverse correlation was observed among states where the prevalence in 1999 exceeded 90% (**Figure 3**). Between-state inequality in ANC service quality increased initially, with IQR doubling between 1999–2006 from 15.6 to 31.7 percentage points, then stabilising until 2016 (IQR = 32.2). However, it declined between 2016–21 (IQR = 18.9), resulting in a slight increase compared to the distribution observed in 1999 (**Figure 4**, Table S12 in the **Online Supplementary Document**). States that performed relatively poorly in terms of ANC quality in 1999 continued to perform poorly in 2006, 2016, and 2021 (Pearson correlation coefficient (r) = 0.890, 0.757, and 0.731; *P* ≤ 0.001), as indicated by the prevalence correlation test and graph showing ranked order between state/UT (Figure S1 and Table S13 in the **Online Supplementary Document**). However, Manipur and Odisha showed relatively greater improvement than other states, falling more than 10 places in ranking between 1999–2021 (from 10th to 27th and from 5th to 19th, respectively).

### Estimated headcount of mothers who received inadequate quality of ANC

We estimate there were approximately 362 865 992 women of childbearing age (15–49 years) throughout India in 2021. Of them, 104 645 059 women – slightly less than one third – did not receive all seven ANC services. The headcounts from Bihar and Uttar Pradesh together made up approximately 46.0% of the total number of women who received inadequate ANC quality throughout India in 2021 (**Table 1**).

**Table 1:** Estimated headcount of inadequate antenatal care for India and 36 states/union territories, and percentage share of inadequate antenatal care of each state/union territory in India, 2021

State/UT	Headcount 2021, n (%)
India*	104 645 059 (100)
Uttar Pradesh	25 864 583 (24.73)
Bihar	22 246 973 (21.27)
Madhya Pradesh	7 773 676 (7.43)
Rajasthan	7 763 673 (7.42)
Jharkhand	5 613 915 (5.37)
Maharashtra	4 969 089 (4.75)
West Bengal	3 843 921 (3.67)
Assam	3 240 810 (3.10)
Gujarat	3 088 272 (2.95)
Odisha	2 981 203 (2.85)
Karnataka	2 653 965 (2.54)
Chhattisgarh	2 559 114 (2.45)
Telangana	1 803 346 (1.72)
Andhra Pradesh	1 737 037 (1.66)
Tamil Nadu	1 387 082 (1.33)
Jammu and Kashmir	1 223 169 (1.17)
Haryana	1 193 687 (1.14)
Punjab	1 179 800 (1.13)
Delhi	776 896 (0.74)
Meghalaya	716 688 (0.69)
Uttarakhand	691 162 (0.66)
Kerala	344 898 (0.33)
Himachal Pradesh	199 395 (0.19)
Tripura	178 413 (0.17)
Nagaland	146 413 (0.14)
Manipur	130 968 (0.13)
Mizoram	105 717 (0.10)
Arunachal Pradesh	88 472 (0.08)
The Dadra, Nagar Haveli, Daman and Diu	29 478 (0.03)
Puducherry	25 451 (0.02)
Chandigarh	24 957 (0.02)
Goa	20 820 (0.02)
Sikkim	16 428 (0.02)
Ladakh	15 899 (0.02)
Andaman and Nicobar	8487 (0.01)
Lakshadweep	1204 (0.00)

UT – union territory

*All-India headcount is the sum of the state/union territory headcount.

**Figure 5:**
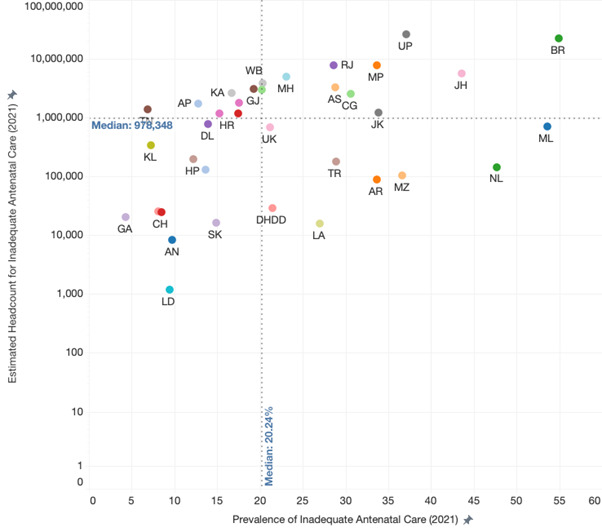
Relationship between the prevalence of inadequate antenatal care and 2021 headcount burden of inadequate antenatal care. AN – Andaman and Nicobar, AP – Andhra Pradesh, AR – Arunachal Pradesh, AS – Assam, BR – Bihar, CH – Chandigarh, CG – Chhattisgarh, DH – Dadra and Nagar Haveli and Daman and Diu, GA – Goa, GJ – Gujarat, HR – Haryana, HP – Himachal Pradesh, JK – Jammu and Kashmir, JH – Jharkhand, KA – Karnataka, KL – Kerala, LK – Ladakh, LD – Lakshadweep, MP – Madhya Pradesh, MH – Maharashtra, MN – Manipur, ML – Meghalaya, MZ – Mizoram, DL – NCT Delhi, NL – Nagaland, OR – Odisha, PY – Puducherry, PB – Punjab, RJ – Rajasthan, S – Sikkim, TN – Tamil Nadu, TL – Telangana, TR – Tripura, UP – Uttar Pradesh, UK – Uttarakhand, WB – West Bengal.

States/UTs with a higher prevalence of inadequate quality ANC contributed more to the total population burden of mothers who received inadequate quality ANC. However, the strength of the correlation was relatively weak (r = 0.479; *P* = 0.003) and lost statistical significance when the states of Uttar Pradesh and Bihar were excluded from the analysis (r = 0.299; *P* = 0.086) (**Figure 5**).

### Distribution of prevalence in mothers who received inadequate quality of ANC by sociodemographic factors 2021

The prevalence of mothers who received inadequate quality of ANC gradually increased as the mother’s education level and household wealth level decreased. The prevalence gap between mothers with the lowest and highest group in education and wealth level amounted to 32.5 and 34.0 percentage points, respectively. Additionally, rural areas exhibited a higher prevalence (32.4%) compared to urban areas (20.1%) (**Table 2**). Disparities in prevalence between women with the lowest and the highest level of education and wealth and between women residing in rural and urban areas were consistently the largest for ultrasound tests (Figure S2 in the **Online Supplementary Document**).

**Table 2:** Sample distribution and percentage (95% confidence interval) of inadequate antenatal care by demographic and socioeconomic characteristics, 2021, India

Characteristics	Sample distribution, n (%)	Inadequate ANC, % (95% CI)
Multiple births		
*No*	130 533 (99.0)	28.9 (28.6–29.2)
*Yes*	1310 (1.0)	23.7 (20.6–26.8)
Sex of child		
*Male*	71 196 (54.0)	28.6 (28.2–29.1)
*Female*	60 647 (46.0)	29.1 (28.6–29.6)
Birth order		
*1*	45 462 (34.5)	21.6 (21.1–22.1)
*2*	48 432 (36.7)	25.7 (25.2–26.3)
*3*	21 826 (16.6)	35.6 (34.8–36.5)
*4*	16 124 (12.2)	49.4 (48.4–50.4)
Pregnant age		
*<20*	13 137 (10.0)	29.5 (28.4–30.6)
*20–29*	97 972 (74.3)	28.3 (27.9–28.7)
*30–34*	15 749 (11.9)	29.8 (28.8–30.8)
*≥35*	4987 (3.8)	34.6 (32.9–36.3)
Highest educational level		
*No education*	24 699 (18.7)	48.2 (47.4–48.9)
*Primary*	15 538 (11.8)	36.6 (35.6–37.7)
*Secondary*	67 821 (51.4)	24.6 (24.2–25.1)
*More than secondary*	23 786 (18.0)	15.7 (15.0–16.5)
*Missing*	0 (0.0)	NA
Wealth quantile		
*Lowest*	27 703 (21.0)	49.1 (48.3–49.8)
*Second*	27 493 (20.9)	33.2 (32.5–33.9)
*Middle*	26 259 (19.9)	24.5 (23.7–25.2)
*Fourth*	26 097 (19.8)	19.9 (19.2–20.7)
*Highest*	24 293 (18.4)	15.1 (14.4–15.8)
Caste		
*Scheduled caste*	29 805 (22.6)	30.9 (30.2–31.6)
*Scheduled tribe*	13 193 (10.0)	35.7 (34.7–36.6)
*Other backward caste*	58 725 (44.5)	28.5 (28.0–29.0)
*Other*	23 664 (17.9)	23.8 (22.9–24.6)
*Missing*	6457 (4.9)	NA
Marital status		
*Never or previously married*	1547 (1.2)	34.5 (31.2–37.8)
*Currently married*	130 296 (98.8)	28.8 (28.4–29.1)
Place of residence		
*Urban*	38 124 (28.9)	20.1 (19.4–20.8)
*Rural*	93 719 (71.1)	32.4 (32.0–32.8)

ANC – antenatal care, CI – confidence interval, NA – not applicable

## DISCUSSION

This study provides the first systematic and comprehensive assessment of ANC quality between 1999–2021 across India and in each of the 36 states/UTs using a composite indicator referred to as ‘inadequate quality of ANC’ (i.e. mothers who did not complete seven essential ANC service items during their ANC visits). A novel approach was employed to account for the evolving state geometries throughout the study period, which enabled comparable state-specific estimations across four survey years. Additionally, we performed multifaceted analyses on this quality indicator, which include examining the long-term prevalence of inadequate quality of ANC, the trend in between-state disparities, the correlation between the initial level of ANC quality and its magnitude of change over the study period at the state level, and estimating the population headcount that did not receive adequate quality of ANC. Understanding the progress and current state of ANC quality is the first step in planning strategies to achieve universal coverage of high-quality services. Given that India, due to its substantial population size, accounts for approximately 17.5% of global maternal deaths, stillbirths, and neonatal deaths [41], achieving the global targets for reducing maternal and neonatal mortality critically depends on India’s progress. Therefore, our research conducted within India holds significant global importance.

Six salient findings emerged from our analyses. First, the overall prevalence of mothers who received inadequate ANC quality, defined as those who did not receive all seven ANC services, decreased from 80.8% to 28.8% between 1999–2021. However, several states still have more than 40% of pregnant mothers who receive inadequate quality of ANC. Second, states with a higher prevalence of inadequate ANC quality in 1999 demonstrated greater improvements by 2021 than states with a lower prevalence of inadequate ANC quality in 1999. Despite this trend, an inverse correlation was observed between initial year prevalence and SAC during the study period among states where the initial prevalence exceeded 90%. Third, we found a slight increase in between-state inequality in the prevalence of inadequate ANC quality over the study period. Fourth, states that performed relatively poorly in terms of ANC quality in 1999 continued to perform poorly in 2021. Fifth, Uttar Pradesh and Bihar, due to their large population size and higher prevalence, jointly represented nearly half of the total population burden associated with inadequate quality of ANC. Finally, substantial disparities exist in the quality of ANC received by pregnant mothers according to several sociodemographic characteristics, notably education and household wealth level.

Our study has several data-related limitations. First, there was a potential for recall bias as mothers were asked to self-report the ANC services they received. However, recent evidence suggests that the validity of ANC (and postnatal care) content indicators is most robust for those pertaining to discrete clinical activities, especially for indicators involving tangible, observable actions such as the administration or omission of specific tests (as opposed to indicators concerning the provision or absence of certain information or advice) [42]. Second, we acknowledge that the seven items used to construct the ANC quality indicator do not comprise the full range of necessary ANC services, and they do not capture the timeliness of the service receipt. Additionally, although certain groups of pregnant mothers with certain conditions (e.g. HIV/AIDS or malaria) may require extra services during pregnancy, we were unable to consider this due to data availability. However, we believe this limitation does not compromise the validity of our findings. A prior study demonstrated a significant association between the composite quality index constructed using these seven ANC services and neonatal mortality [37]. Furthermore, several studies have also used a subset of these seven services as a proxy for ANC quality. Third, while it is advised that certain ANC services, such as weight and blood pressure measurement, be provided at each ANC visit, the NFHS only investigated whether mothers received these services at least once at any point throughout the entire pregnancy. Fourth, although coronavirus disease 2019 (COVID-19) may have disrupted data collection and the provision and uptake of health care services during NFHS-5, we have not comprehensively examined any potential systematic influence of the pandemic on our results. While there is abundant research on the effect of COVID-19 on ANC utilisation [43–45], studies specifically addressing its impact on ANC quality are limited. It is anticipated that COVID-19 negatively affected ANC quality because restrictions on face-to-face contact might have hindered conducting diagnostic tests and the transport of samples to laboratories. However, these negative impacts might vary by socioeconomic status or geographical areas, including states or communities. Fifth, due to changes in state geometry during the study period, estimates had to be approximated for the states of Andhra Pradesh, Bihar, Chhattisgarh, Jharkhand, Madhya Pradesh, Telangana, Uttar Pradesh, Uttarakhand, and Jammu and Kashmir in 1999, and for the states of Andhra Pradesh, Telangana, Jammu and Kashmir, and Ladakh in 2006 using the approaches validated in a previous study. Finally, we intentionally excluded ANC received at home, as our research aimed to examine ANC quality within the formal health system. While this approach is not a limitation, it is important to recognise that our results do not represent the entire spectrum of ANC quality in India. Home-based ANC services are generally considered to be lower quality than those provided in health facilities due to several potential issues, such as the frequent unavailability of necessary equipment or the lower skill level of home-visit health workers [46,47]. Consequently, the absolute prevalence and headcount of inadequate ANC quality would likely be higher if these groups were included. On the other hand, as the proportion of home-based ANC has been decreasing, the reduction rate in inadequate ANC quality would likely be more pronounced if these groups were considered.

Substantial policies for enhancing health service quality have been implemented both at the national level and within specific states in India. Although their primary focus may not have been on addressing service quality issues, these policy initiatives are believed to have played a significant role in reducing the proportion of women receiving inadequate quality ANC by one-third over the past 22 years. However, over 25% of women in our sample still do not receive all seven of the most essential ANC services as of 2021. Earlier studies have documented that even among women who initiate ANC in the first trimester and attend more than four ANC visits, the content of ANC provided remained suboptimal [48]. This collective evidence indicates that more quality-focused policies are required.

We observed a slight increase in between-state disparities between 1999–2021 in inadequate quality of ANC despite a substantial overall decline in prevalence. The proportion of women receiving inadequate quality ANC varied greatly from state to state, ranging from 4.3% in Goa to 55.0% in Bihar as of 2021. Several factors likely contributed to these increased disparities. Socioeconomic determinants, which have long been identified as primary drivers of health service utilisation and quality in previous studies, may have played a significant role [49,50]. Although the economy in India is rapidly expanding, the growth has been uneven; certain states experienced more robust development, which facilitated better investment in health care infrastructure, while others have lagged behind [51]. Furthermore, evidence suggests a gap in educational attainment between states has widened [52]. The varying effectiveness of health programs, potentially due to differences in governance and administrative capabilities, may also have contributed to the increased disparities [51].

We also found that the prevalence of the specific ANC service items varied across states. For example, in 2021, far fewer women in Arunachal Pradesh received all seven ANC services than women in Bihar (33.9% vs 54.9%, respectively), but the prevalence of women having not taken tetanus injections was much higher (11.7% in Arunachal Pradesh vs 3.1% in Bihar). Similarly, while Bihar and Meghalaya states showed similar prevalence of non-receipt of all seven ANC services (54.9% and 53.4%, respectively), there was a big difference in the proportion of women who have not taken iron (22.3% in Bihar vs 7.2% in Meghalaya). Inequalities in ANC quality by socioeconomic status (SES) were also still substantial in 2021. The prevalence of inadequate quality of ANC among women from the lowest education and wealth groups was more than three times higher compared to those from the highest education and wealth groups (48.2% vs 15.7% for educational level and 49.1% vs 15.1% for wealth level). These findings underscore the need for approaches that are both geographically and socioeconomically targeted in terms of the provision of ANC services.

We found that some states still lag despite large-scale policy efforts. For instance, the Bill and Melinda Gates Foundations, in partnership with the Government of Bihar, launched the Ananya program in 2010 to improve the quality of maternal, newborn and child health and nutrition services in Bihar. However, the program’s approach regarding ANC primarily focused on enhancing outreach by frontline health workers rather than strengthening ANC capacity of the providers at health facilities, which may have contributed to Bihar’s ongoing challenges in delivering essential ANC services [53]. Although the Ananya program promoted the consumption of iron-folic acid tablets for 90 days or more – one of the seven ANC items we considered in our analyses – Bihar still recorded the second highest rate of non-consumption of iron supplements in 2021. Policies need to be carefully appraised in terms of the impact on enhancing the health care service quality.

As expected, low SES correlated with inadequate quality of ANC. Several mechanisms might contribute to this correlation. From the user side, out-of-pocket costs often hinder accessing ANC services. Although ANC is officially free in many low- and middle-income countries, consultations often involve user charges for specific services or consumables [54]. Additionally, women with low levels of education and empowerment may not recognise what constitutes high-quality services [55], and therefore, they are less likely to complain about poor-quality care or request better services [56]. From the provider and system side, a lack of provider capacity and the necessary infrastructure for adequate quality of ANC are more common in disadvantaged communities [57], resulting in a higher prevalence of low-quality ANC among lower SES women.

The most pronounced disparities between different socioeconomic groups were observed in the prevalence of not receiving ultrasound tests, followed by the prevalence of not receiving urine and blood tests. The gaps in the prevalence of not receiving tetanus injections were relatively small. The low rate of receiving service items requiring laboratory tests can be linked to non-compliance among mothers who cannot afford user fees, as previously mentioned [54]. It was reported that charges are particularly common for booking appointments, medicines, laboratory tests, and ultrasound scans [58,59]. The lack of laboratory facilities, supplies, and ultrasound equipment in disadvantaged communities also contributes to the large disparities in the prevalence of not receiving urine tests and blood tests [57] Conversely, the relatively minor gap in the prevalence of not receiving tetanus injections is likely due to the widespread availability of free vaccination services. Policy interventions must encompass efforts aimed at ensuring provider and system capacity to deliver essential services and removing financial barriers for users to ensure access to these services.

## CONCLUSIONS

Access to high-quality care is imperative in achieving the SDG for maternal and neonatal mortality. Future research should focus on how we can accelerate enhancements in perinatal service quality and effectively address disparities. This may include exploring novel approaches regarding health care worker training programs and developing robust quality assurance mechanisms. Investigating the potential of digital health technologies and integrating multi-dimensional approaches encompassing structural readiness and financial protection mechanisms in quality improvement is necessary. Additionally, research on effectively targeting vulnerable segments of the population in India is required. These efforts would provide valuable insights for policy and practice to improve the quality of perinatal service, including ANC, in India.

## Additional material


Online Supplementary Document
