# Supplementary material for: Trends in the quality of antenatal care in India: Patterns of change across 36 states and union territories, 1999–2021
Source: J Glob Health. 2024 Oct 18;14:04188. doi: 10.7189/jogh.14.04188 (PMC11487464; doi:10.7189/jogh.14.04188)

Table S1. Illustrative list of national and selected state policies related to ANC service quality in India

Policy	Launched	Purpose
**Nationwide or across multiple states**		
Child Survival and Safe Motherhood Programme	1992	This program aims to reduce maternal and child mortality. There was a focus placed on antenatal care as a part of this program via the training of Traditional Birth Attendants to provide ANC services. ANC quality was addressed with increased training of providers, improving supply chain management, and implementing monitoring & evaluation.
Empowered Action Group (EAG)	2001	This Initiative by the central government aims to organize eight low-performing states (Bihar, Chhattisgarh, Jharkhand, Madhya Pradesh, Odisha, Rajasthan, Uttarakhand, and Uttar Pradesh) in order to improve maternal and child health outcomes. The provision of ANC services was a core component of this. ANC quality is addressed in part through the Mother and Child Tracking System. This digital tool is used by providers to track how many ANC visits a mother has attended, and which ANC services she has received.
National Rural Health Mission (NRHM)	2005	This program aims to address the needs of underserved rural areas throughout India. One major initiative was the creation of Accredited Social Health Activists (ASHAs), local health workers who focused on the health needs of women and children. ASHAs are expected to provide a whole host of health services to beneficiaries, including ANC. Quality of care is addressed through trainings. These trainings cover all aspects of ANC care delivery and highlight the importance of visiting mothers at home as well as seeing them at the health center.
Reproductive, maternal, newborn, child and adolescent health (RMNCH+A)	2013	A strategy was designed to improve maternal and child health outcomes by integrating interventions across the life course. This strategy includes guidance on improving ANC quality of care through "Skills Labs", training sessions for providers. Skill trainings focus on ensuring ANC providers know the expected date of delivery, how to check blood pressure, and how to weigh-ins.
Dakshata	2015	This initiative strengthens the quality of care during and immediately after childbirth by using focused and customized trainings for providers (medical officers, nurses, and auxiliary nurse midwifes). This program has developed comprehensive checklists to be completed by ANC providers to ensure that they have provided all the components of ANC services.
Pradhan Mantri Surakshit Matritva Abhiyan	2016	PMSMA guarantees a minimum package of high-quality antenatal care services to women in their 2nd and 3rd trimesters at designated government health facilities on the 9th of every month. Quality, in this case, is largely addressed through ensuring that there is sufficient person-power at each PMSMA site. PMSMA says there needs to be 2 staff nurses, 2 lab technicians, 3-4 ANMs, and 2 counsellors for every 100 pregnant women attending a PMSMA clinic.
POSHAN Abhiyaan	2018	POSHAN Abhiyaan is a comprehensive nation-wide nutrition program. This program helps improve ANC quality by emphasizing the need for growth monitoring and IFA supplementation during the antenatal period. Provision of IFA and growth monitoring have to be monitored in order of POSHAN Abhiyan targets to be met.
Anemia Mukt Bharat	2018	This comprehensive program aims to reduce the burden of anemia throughout the country. Iron and folic acid (IFA) supplementation along with blood testing at ANCs is a component of this program and must be monitored for targets to be met.
Bihar		

Bihar Technical Support Program with CARE	2011	This is a multidimensional program implemented in partnership between CARE, BTSP, and Bihar state government to improve maternal & child health in the state. Improving ANC quality was a part of this program. More specifically, this training utilizes the incremental learning approach to offer training, practice, and supportive supervision relating to the various components of ANC care.
Odisha		
Mobile Health Units (MHU)	1995	These mobile health units provide various forms of health care services to people living in last-mile communities, including ANC services. This improves quality by ensuring the provision of IFA and blood checks to woman coming for ANC.
Tamil Nadu		
Pregnancy Infant Cohort Monitoring and Evaluation (PICME)	2009	PICME is a digital tracking system to assess what care services have been utilized by women, including ANC services. By tracking which women have received which services, providers can fill gaps in service provision to make sure that women have received all of the ANC services.
Uttar Pradesh		
Uttar Pradesh Technical Support Unit	2013	Like the BTSP, this unit works with the UP state government to improve uptake and quality of ANC services throughout the state. The effort focuses on provider training and capacity building.
Uttarakhand		
Uttarakhand state public health policy	2020	This covers all aspects of health within the state along with an emphasis on improving quality and uptake of ANC services. In terms of quality of services, the policy focuses on provider training, outlines treatment protocols and case management guidelines, and has developed a data management system that allows providers insights into patients to see what services they have received and what they need.
Haryana		
State government partnership with WHO to improve quality of care in three districts	2017	This intervention focuses on: 1) infrastructural gaps of the clinics where ANC services are delivered, 2) improving patient support facilities, 3) improving clinical practices, 4) provider capacity building, 5) case documentation, 6) supervision & monitoring, and 7) communication. Overall, this intervention led to improved patient satisfaction

Table S2. Analytic sample characteristics (weighted), India: 1999, 2006, 2016, and 2021.

Sociodemographic variables	Sociodemographic variables	1999 (N=15,061)	2006 (N=23,137)	2016 (N=127,515)	2021 (N=131,842)
Multiple birth	no	14954 (99.3%)	22921 (99.1%)	126343 (99.1%)	130533 (99.0%)
	yes	108 (0.7%)	217 (0.9%)	1173 (0.9%)	1310 (1.0%)
Sex of child	male	8107 (53.8%)	12507 (54.1%)	69516 (54.5%)	71196 (54.0%)
	female	6955 (46.2%)	10631 (45.9%)	58000 (45.5%)	60647 (46.0%)
Birth order	1	5047 (33.5%)	7138 (30.8%)	45561 (35.7%)	45462 (34.5%)
	2	4542 (30.2%)	7531 (32.5%)	46036 (36.1%)	48432 (36.7%)
	3	2622 (17.4%)	3894 (16.8%)	20270 (15.9%)	21826 (16.6%)
	4	2852 (18.9%)	4576 (19.8%)	15649 (12.3%)	16124 (12.2%)
Pregnant age (yrs.)	<20	3549 (23.6%)	4758 (20.6%)	15159 (11.9%)	13137 (10.0%)
	20-29	9842 (65.3%)	15519 (67.1%)	94773 (74.3%)	97972 (74.3%)
	30-34	1238 (8.2%)	2129 (9.2%)	13073 (10.3%)	15749 (11.9%)
	>=35	435 (2.9%)	734 (3.2%)	4512 (3.5%)	4987 (3.8%)
Highest educational level	no education	5709 (37.9%)	8371 (36.2%)	29181 (22.9%)	24699 (18.7%)
	primary	1462 (9.7%)	1900 (8.2%)	7186 (5.6%)	15538 (11.8%)
	secondary	5749 (38.2%)	10978 (47.4%)	73650 (57.8%)	67821 (51.4%)
	higher	2136 (14.2%)	1889 (8.2%)	17500 (13.7%)	23786 (18.0%)
	missing	7 (0.0%)	2 (0.0%)	-	-
Wealth quintile	lowest quintile (=the poorest)	2032 (13.5%)	3976 (17.2%)	23072 (18.1%)	27703 (21.0%)
	second quintile	2605 (17.3%)	4407 (19.0%)	26215 (20.6%)	27493 (20.9%)
	middle quintile	2873 (19.1%)	4810 (20.8%)	27382 (21.5%)	26259 (19.9%)
	fourth quintile	3658 (24.3%)	5016 (21.7%)	26663 (20.9%)	26097 (19.8%)
	highest quintile (=the wealthiest)	3896 (25.9%)	4930 (21.3%)	24184 (19.0%)	24293 (18.4%)
Caste	SC	2665 (17.7%)	4408 (19.0%)	26256 (20.6%)	29805 (22.6%)
	ST	1072 (7.1%)	1879 (8.1%)	12800 (10.0%)	13193 (10.0%)
	OBC	4357 (28.9%)	9013 (39.0%)	56855 (44.6%)	58725 (44.5%)
	Others	6863 (45.6%)	7195 (31.1%)	26145 (20.5%)	23664 (17.9%)
	missing	107 (0.7%)	645 (2.8%)	5462 (4.3%)	6457 (4.9%)
Marital status	never married or previously married	170 (1.1%)	362 (1.6%)	1673 (1.3%)	1547 (1.2%)
	currently married	14892 (98.9%)	22776 (98.4%)	125843 (98.7%)	130296 (98.8%)
Place of residence	urban	5190 (34.5%)	7858 (34.0%)	41046 (32.2%)	38124 (28.9%)
	rural	9872 (65.5%)	15280 (66.0%)	86470 (67.8%)	93719 (71.1%)

Table S3. The prevalence of mothers who have received inadequate quality of ANC and 95% confidence interval for India and 36 states/union territories,1999 -2021

<10 | 10≤&<40 | 40≤&<70 | 70≤&<90 | ≥90

	1999			2006			2016			2021		
	%	LCI	UCI	%	LCI	UCI	%	LCI	UCI	%	LCI	UCI
**All-India**	**84.8**	**84.1**	**85.5**	**73.9**	**73.2**	**74.6**	**43.4**	**42.9**	**43.7**	**28.8**	**28.5**	**29.2**
Andhra Pradesh	84.0	79.3	87.8	55.9	52.3	59.5	14.2	12.7	15.7	12.8	11.3	14.5
Arunachal Pradesh	97.6	94.3	99.0	81.4	75.4	86.3	55.8	53.1	58.4	33.6	31.7	35.6
Assam	97.7	96.2	98.6	90.0	87.5	92.0	53.4	52.0	54.7	28.8	27.6	30.1
Bihar	95.5	93.1	97.1	87.6	84.1	90.4	74.2	73.1	75.3	55.0	53.8	56.1
Chhattisgarh	96.2	91.2	98.4	92.3	90.4	93.8	48.5	46.9	50.1	30.6	29.1	32.2
Goa	44.4	38.7	50.2	26.5	22.1	31.4	9.5	6.4	13.7	4.3	2.5	7.5
Gujarat	75.8	72.1	79.1	67.3	63.9	70.5	33.0	31.2	34.9	19.3	18.0	20.6
Haryana	83.8	80.2	86.9	70.2	64.2	75.6	24.5	23.0	26.0	15.3	14.2	16.4
Himachal Pradesh	88.0	85.2	90.4	62.0	57.1	66.6	20.0	17.6	22.7	12.2	10.1	14.7
Jharkhand	97.8	94.2	99.2	91.5	89.3	93.3	64.3	62.9	65.6	43.6	42.3	45.0
Karnataka	79.9	76.8	82.8	53.6	50.8	56.3	22.2	20.6	23.9	16.7	15.4	18.0
Kerala	62.3	57.9	66.4	28.8	24.7	33.4	5.9	4.4	7.7	7.2	5.7	9.1
Madhya Pradesh	93.1	91.3	94.6	87.3	85.0	89.2	50.7	49.6	51.8	33.7	32.6	34.8
Maharashtra	71.6	68.6	74.4	52.1	49.4	54.8	26.8	24.9	28.8	23.1	20.9	25.4
Manipur	94.5	92.0	96.3	80.3	77.9	82.5	27.9	26.3	29.5	13.7	12.2	15.3
Meghalaya	94.4	90.8	96.7	87.9	84.8	90.5	66.5	64.4	68.5	53.6	51.3	55.9
Mizoram	94.5	91.1	96.7	91.8	88.7	94.1	51.3	48.2	54.4	36.7	33.2	40.3
Nagaland	99.0	96.2	99.8	94.1	92.1	95.6	68.4	65.5	71.1	47.7	44.6	50.8
Odisha	97.4	96.1	98.3	87.6	85.0	89.7	40.3	39.0	41.6	20.2	19.1	21.4
Punjab	82.6	79.2	85.6	61.0	55.6	66.1	16.6	14.9	18.6	17.5	15.9	19.1
Rajasthan	91.9	89.9	93.5	84.5	82.0	86.7	47.9	46.9	49.0	28.6	27.6	29.7
Sikkim	92.5	88.5	95.1	63.0	58.2	67.5	10.3	8.1	13.1	14.8	10.5	20.6
Tamil Nadu	67.9	63.6	72.0	48.3	45.0	51.6	28.7	27.1	30.3	6.9	6.0	7.8
Telangana	73.1	67.8	77.9	55.9	52.3	59.5	15.2	13.3	17.4	17.5	16.2	18.9
Tripura	93.1	88.3	96.1	84.2	80.2	87.5	54.0	50.1	57.9	28.9	25.4	32.6
Uttar Pradesh	93.7	92.0	95.1	92.5	91.3	93.6	70.4	69.6	71.1	37.1	36.3	37.9
Uttarakhand	88.3	80.2	93.4	70.7	65.4	75.5	43.0	1.1	40.8	21.2	19.0	23.5
West Bengal	93.9	92.3	95.1	85.1	83.2	86.9	41.7	39.3	44.2	20.3	18.6	22.1
Delhi	65.1	61.1	68.9	44.0	40.1	48.0	18.4	15.2	22.1	13.9	12.3	15.7
Jammu & Kashmir (UT)	95.8	94.2	97.0	86.6	83.8	88.9	37.4	35.8	39.1	33.8	32.2	35.5
Ladakh (UT)				86.6	83.8	88.9	31.3	27.1	35.9	27.0	22.8	31.6
Andaman & Nicobar (UT)							13.8	10.0	18.7	9.8	6.7	14.0
Chandigarh (UT)							22.0	14.6	31.8	8.5	4.6	15.2
Dadra Nagar Haveli and Daman & Diu (UT)							35.9	29.6	42.8	21.5	17.2	26.4
Lakshadweep (UT)							7.4	3.9	13.7	9.4	5.4	15.9
Puducherry (UT)							19.3	15.1	24.5	8.1	4.9	13.3

Table S4. Absolute change in the prevalence of inadequate quality of ANC between 1999-2006, 2006-2016, 2016-2021, and 1999-2021 for India and 36 states/union territories

**<-40**	**-39.9 to -20**	**-19.9 to -10**	**-9.9 to -0.1**	**>0**

	1999-2021	1999-2006	2006-2016	2016-2021
Andhra Pradesh	-71.21	-28.09	-41.75	-1.36
Arunachal Pradesh	-63.98	-16.19	-25.67	-22.12
Assam	-68.86	-7.69	-36.61	-24.56
Bihar	-40.57	-7.95	-13.40	-19.22
Chhattisgarh	-65.60	-3.94	-43.77	-17.89
Goa	-40.07	-17.94	-17.01	-5.12
Gujarat	-56.52	-8.51	-34.29	-13.71
Haryana	-68.56	-13.60	-45.72	-9.24
Himachal Pradesh	-75.83	-26.04	-41.99	-7.80
Jharkhand	-54.14	-6.28	-27.22	-20.64
Karnataka	-63.29	-26.35	-31.38	-5.56
Kerala	-55.03	-33.43	-22.98	1.38
Madhya Pradesh	-59.41	-5.85	-36.60	-16.97
Maharashtra	-48.46	-19.45	-25.32	-3.69
Manipur	-80.84	-14.21	-52.42	-14.20
Meghalaya	-40.85	-6.52	-21.40	-12.93
Mizoram	-57.89	-2.72	-40.50	-14.67
Nagaland	-51.35	-4.95	-25.72	-20.68
Odisha	-77.25	-9.88	-47.26	-20.10
Punjab	-65.15	-21.65	-44.35	0.85
Rajasthan	-63.26	-7.44	-36.53	-19.29
Sikkim	-77.63	-29.46	-52.69	4.52
Tamil Nadu	-61.07	-19.64	-19.61	-21.81
Telangana	-55.60	-17.20	-40.72	2.31
Tripura	-64.28	-8.98	-30.11	-25.18
Uttar Pradesh	-56.67	-1.20	-22.19	-33.29
Uttarakhand	-67.18	-17.66	-27.68	-21.84
West Bengal	-73.58	-8.75	-43.37	-21.46
Andaman & Nicobar				-4.03
Chandigarh				-13.55
Delhi	-51.14	-21.07	-25.61	-4.46
The Dadra Nagar Haveli, Daman & Diu				-14.46
Jammu & Kashmir	-61.98	-9.23	-49.15	-3.60
Ladakh			-55.23	-4.36
Lakshadweep				1.99
Puducherry				-11.23

Table S5. The prevalence of mothers who have not been weighed and 95% confidence interval for India and 36 states/union territories,1999 -2021

State	1999			2006			2016			2021		
	%	LCI	UCI	%	LCI	UCI	%	LCI	UCI	%	LCI	UCI
**India**	40.9	39.9	41.9	33.0	32.2	33.8	9.4	9.2	9.6	2.8	2.7	3.0
**States**												
Andhra Pradesh	24.0	19.3	29.3	8.2	6.4	10.5	0.6	0.3	1.0	0.1	0.0	0.4
Arunachal Pradesh	36.2	30.0	42.9	17.7	12.9	23.7	3.7	2.9	4.8	2.7	2.2	3.4
Assam	71.0	66.9	74.7	49.6	45.5	53.7	1.8	1.5	2.2	1.5	1.2	1.8
Bihar	60.6	55.9	65.2	47.5	42.2	52.8	21.3	20.3	22.4	8.9	8.3	9.6
Chhattisgarh	58.1	49.5	66.3	41.3	37.8	44.9	2.3	1.8	2.8	0.8	0.6	1.2
Goa	13.5	10.0	18.0	1.9	0.8	4.1	0.4	0.1	2.6	0.0	0.0	0.0
Gujarat	30.7	27.1	34.6	25.4	22.5	28.6	2.3	1.8	2.9	0.5	0.3	0.8
Haryana	18.6	15.3	22.4	37.8	32.0	43.9	3.1	2.6	3.8	0.9	0.6	1.2
Himachal Pradesh	35.8	31.8	40.0	13.5	10.4	17.3	6.1	4.9	7.7	1.1	0.6	2.0
Jharkhand	53.8	46.6	60.9	44.3	40.3	48.4	8.1	7.4	8.9	3.4	3.0	3.9
Karnataka	29.0	25.7	32.5	17.5	15.5	19.7	0.9	0.6	1.5	0.4	0.2	0.7
Kerala	19.5	16.3	23.3	4.8	3.1	7.3	0.5	0.2	1.3	0.7	0.3	1.5
Madhya Pradesh	65.1	62.0	68.1	41.6	38.5	44.9	5.3	4.9	5.8	1.4	1.1	1.7
Maharashtra	26.9	23.9	30.2	7.9	6.5	9.6	1.4	1.0	1.9	0.8	0.6	1.2
Manipur	42.0	37.5	46.6	15.3	13.2	17.5	2.7	2.2	3.4	1.2	0.7	1.9
Meghalaya	40.4	34.5	46.6	14.0	11.0	17.7	2.0	1.4	2.7	1.8	1.3	2.5
Mizoram	24.1	19.2	29.8	11.9	9.1	15.4	3.2	2.4	4.2	2.2	1.4	3.4
Nagaland	91.9	87.2	95.0	57.4	53.6	61.0	16.2	14.1	18.5	4.7	3.6	6.1
Odisha	48.4	44.8	52.0	35.3	31.7	39.0	1.6	1.3	2.0	0.4	0.2	0.6
Punjab	20.9	17.6	24.6	32.5	27.7	37.8	2.9	2.0	4.0	1.3	0.9	1.9
Rajasthan	63.9	60.6	67.0	50.2	46.9	53.6	7.5	6.9	8.1	2.3	1.9	2.6
Sikkim	64.1	58.2	69.6	18.0	14.4	22.2	0.1	0.0	0.6	0.3	0.0	1.8
Tamil Nadu	13.9	11.0	17.5	2.6	1.8	3.9	0.3	0.2	0.5	0.1	0.0	0.2
Telangana	13.4	9.9	17.8	8.2	6.4	10.5	0.7	0.4	1.2	0.2	0.1	0.4
Tripura	42.5	35.6	49.8	27.7	23.4	32.5	2.1	1.3	3.4	1.6	0.9	2.7
Uttar Pradesh	68.5	65.5	71.4	75.7	73.8	77.6	34.2	33.4	34.9	5.8	5.4	6.2
Uttarakhand	56.6	46.9	65.8	39.5	34.1	45.1	14.0	12.6	15.5	2.5	1.7	3.7
West Bengal	36.2	32.8	39.7	18.9	16.8	21.1	1.2	0.7	1.9	0.7	0.4	1.2
**Union territories**												
NCT Delhi	10.4	8.2	13.2	8.6	6.7	10.9	3.4	2.1	5.2	1.2	0.7	1.8
Jammu & Kashmir	77.5	74.5	80.3	73.8	70.4	77.0	12.7	11.7	13.7	1.9	1.5	2.4
Ladakh				73.8	70.4	77.0	5.2	3.0	8.9	1.4	0.6	3.2
Andaman & Nicobar							0.2	0.0	1.4	0.0	NA	NA
Chandigarh							3.2	1.0	9.4	0.0	NA	NA
D & N.H & D.D**							2.2	1.0	5.1	0.2	0.0	1.6
Lakshadweep							0.0	0.0	0.0	0.0	NA	NA
Puducherry							0.3	0.1	1.7	0.0	NA	NA

Table S6. The prevalence of mothers who have not received blood pressure check and 95% confidence interval for India and 36 states/union territories,1999 -2021

State	1999			2006			2016			2021		
	%	LCI	UCI	%	LCI	UCI	%	LCI	UCI	%	LCI	UCI
**India**	32.4	31.5	33.3	32.2	31.4	33.0	10.4	10.2	10.6	3.7	3.5	3.8
**States**												
Andhra Pradesh	10.5	7.4	14.7	7.9	6.1	10.2	1.0	0.7	1.5	0.3	0.1	0.8
Arunachal Pradesh	22.4	17.4	28.5	15.3	10.9	21.2	4.3	3.4	5.5	2.5	2.0	3.2
Assam	39.7	35.5	44.1	44.0	39.9	48.1	2.9	2.5	3.4	2.2	1.9	2.7
Bihar	37.1	32.6	41.8	25.6	21.1	30.6	26.5	25.4	27.7	12.3	11.6	13.1
Chhattisgarh	54.3	45.7	62.6	57.5	53.9	61.0	5.4	4.7	6.2	1.0	0.7	1.4
Goa	1.4	0.5	3.6	1.8	0.8	4.0	0.7	0.2	3.2	0.0	NA	NA
Gujarat	26.9	23.4	30.7	22.5	19.7	25.6	2.9	2.4	3.5	0.6	0.4	0.8
Haryana	23.5	19.9	27.6	42.8	36.9	49.0	3.0	2.5	3.6	1.0	0.7	1.3
Himachal Pradesh	36.9	32.9	41.1	18.7	15.1	23.0	4.2	3.1	5.5	0.8	0.4	1.6
Jharkhand	42.0	35.1	49.3	38.8	34.9	42.8	12.7	11.8	13.6	5.4	4.8	6.0
Karnataka	18.6	15.9	21.7	9.0	7.5	10.6	0.8	0.5	1.3	0.5	0.3	1.0
Kerala	3.2	1.9	5.2	1.4	0.6	3.2	0.8	0.4	1.7	0.8	0.4	1.6
Madhya Pradesh	56.9	53.8	60.0	53.8	50.6	57.0	8.7	8.2	9.4	1.9	1.6	2.3
Maharashtra	25.5	22.5	28.7	10.4	8.8	12.2	2.0	1.6	2.6	1.5	1.2	2.0
Manipur	20.0	16.5	23.9	8.0	6.5	9.8	1.2	0.9	1.6	1.1	0.7	1.9
Meghalaya	23.5	18.6	29.2	15.6	12.5	19.4	2.1	1.5	2.8	1.1	0.8	1.4
Mizoram	16.0	12.0	21.1	19.9	16.3	24.0	3.4	2.6	4.5	1.2	0.7	2.2
Nagaland	18.7	13.9	24.6	27.8	24.5	31.4	8.5	7.0	10.3	3.2	2.4	4.4
Odisha	43.7	40.2	47.3	46.2	42.4	50.0	3.1	2.7	3.6	1.0	0.7	1.4
Punjab	15.8	12.9	19.2	20.4	16.4	25.1	1.7	1.1	2.8	0.5	0.3	0.8
Rajasthan	46.8	43.5	50.1	50.1	46.7	53.5	9.9	9.3	10.6	3.1	2.7	3.6
Sikkim	33.2	27.9	39.0	15.2	11.9	19.3	0.6	0.2	1.7	1.7	0.8	3.8
Tamil Nadu	14.5	11.5	18.2	4.8	3.6	6.4	0.3	0.2	0.5	0.1	0.0	0.2
Telangana	7.7	5.1	11.4	7.9	6.1	10.2	0.6	0.3	1.0	0.3	0.1	0.4
Tripura	20.5	15.2	26.9	18.7	15.0	23.1	1.5	0.8	2.6	2.0	1.2	3.2
Uttar Pradesh	58.7	55.5	61.8	69.8	67.7	71.8	33.6	32.9	34.4	6.7	6.3	7.1
Uttarakhand	56.7	46.9	65.9	31.1	26.1	36.6	13.0	11.7	14.5	2.8	2.0	3.9
West Bengal	35.5	32.1	39.1	27.4	25.0	29.9	2.3	1.7	3.3	1.2	0.8	1.8
**Union territories**												
NCT Delhi	11.9	9.4	14.8	10.8	8.7	13.4	2.6	1.5	4.2	1.3	0.9	2.1
Jammu & Kashmir	19.1	16.4	22.0	17.1	14.4	20.2	3.7	3.2	4.3	1.3	1.0	1.6
Ladakh				17.1	14.4	20.2	2.7	1.2	6.0	1.3	0.5	3.0
Andaman & Nicobar							0.3	0.1	1.3	0.0	NA	NA
Chandigarh							2.2	0.5	8.3	0.0	NA	NA
D & N.H & D.D**							3.9	2.0	7.6	0.2	0.0	1.6
Lakshadweep							0.0	0.0	0.0	0.0	NA	NA
Puducherry							0.3	0.1	1.5	0.0	0.0	0.1

Table S7. The prevalence of mothers who have not taken urine test and 95% confidence interval for India and 36 states/union territories,1999 -2021

State	1999			2006			2016			2021		
	%	LCI	UCI	%	LCI	UCI	%	LCI	UCI	%	LCI	UCI
**India**	40.5	39.6	41.5	38.4	37.6	39.2	12.1	11.8	12.3	6.5	6.3	6.7
**States**												
Andhra Pradesh	24.7	20.0	30.1	9.1	7.2	11.4	1.5	1.0	2.1	0.7	0.4	1.3
Arunachal Pradesh	39.0	32.6	45.7	28.2	22.3	35.0	16.3	14.5	18.4	4.0	3.3	4.9
Assam	59.4	55.1	63.6	58.5	54.5	62.5	10.6	9.8	11.5	5.1	4.5	5.8
Bihar	41.9	37.3	46.7	37.7	32.7	43.1	28.7	27.6	29.9	19.4	18.5	20.3
Chhattisgarh	58.8	50.2	66.9	65.1	61.6	68.4	11.4	10.4	12.4	3.7	3.1	4.4
Goa	6.9	4.5	10.5	2.0	0.9	4.1	0.7	0.2	3.1	0.9	0.2	3.4
Gujarat	34.5	30.7	38.5	32.5	29.3	35.9	5.8	5.1	6.7	1.3	1.0	1.7
Haryana	31.0	27.0	35.3	40.1	34.3	46.3	5.7	5.0	6.5	3.2	2.7	3.8
Himachal Pradesh	36.1	32.1	40.3	18.7	15.1	23.0	3.4	2.4	4.8	1.5	0.9	2.5
Jharkhand	48.0	40.8	55.2	52.0	48.0	56.0	19.0	17.9	20.1	10.9	10.1	11.7
Karnataka	31.5	28.2	35.1	13.1	11.4	15.1	0.8	0.6	1.1	0.6	0.4	0.9
Kerala	2.5	1.4	4.4	1.2	0.5	2.9	0.2	0.1	0.6	0.8	0.4	1.6
Madhya Pradesh	55.8	52.7	59.0	56.5	53.3	59.7	12.5	11.8	13.2	5.1	4.6	5.7
Maharashtra	31.3	28.1	34.6	14.4	12.5	16.5	2.2	1.7	2.9	2.1	1.6	2.7
Manipur	60.0	55.3	64.4	27.3	24.7	30.0	6.9	6.0	7.8	2.3	1.7	3.1
Meghalaya	60.5	54.3	66.4	43.2	38.6	47.8	16.9	15.2	18.7	7.0	5.9	8.1
Mizoram	49.4	43.3	55.4	49.5	44.7	54.3	17.8	15.8	20.0	10.6	8.8	12.8
Nagaland	84.9	79.1	89.3	60.5	56.8	64.0	28.1	25.4	30.9	11.9	10.1	14.0
Odisha	57.8	54.2	61.2	49.9	46.1	53.7	6.4	5.7	7.1	2.4	1.9	2.9
Punjab	22.6	19.1	26.4	20.4	16.4	25.1	2.4	1.7	3.4	1.5	1.0	2.2
Rajasthan	49.9	46.6	53.2	52.1	48.7	55.4	11.5	10.8	12.2	6.1	5.6	6.7
Sikkim	45.6	39.8	51.5	20.8	17.0	25.2	1.2	0.6	2.5	2.8	1.1	6.6
Tamil Nadu	19.5	16.0	23.4	6.3	4.9	8.1	0.3	0.2	0.5	0.1	0.0	0.3
Telangana	16.4	12.5	21.1	9.1	7.2	11.4	0.5	0.3	1.0	0.3	0.2	0.6
Tripura	40.9	34.0	48.2	35.0	30.3	40.0	15.3	12.7	18.4	3.1	2.1	4.7
Uttar Pradesh	59.4	56.2	62.5	71.3	69.2	73.2	32.5	31.8	33.3	11.2	10.7	11.7
Uttarakhand	46.5	37.3	56.1	29.3	24.4	34.7	17.3	15.7	19.0	5.6	4.4	7.0
West Bengal	59.3	55.8	62.7	48.6	45.9	51.3	5.3	4.3	6.5	2.4	1.8	3.1
**Union territories**												
NCT Delhi	12.1	9.6	15.0	11.0	8.9	13.6	4.3	2.7	6.6	3.9	3.0	4.9
Jammu & Kashmir	17.0	14.5	19.9	10.2	8.1	12.8	2.8	2.3	3.3	0.7	0.5	1.0
Ladakh				10.2	8.1	12.8	0.8	0.3	2.2	0.5	0.1	2.1
Andaman & Nicobar							0.4	0.1	1.6	0.3	0.1	1.3
Chandigarh							1.0	0.1	7.1	0.0	NA	NA
D & N.H & D.D**							6.0	3.6	9.8	0.6	0.2	1.7
Lakshadweep							0.0	0.0	0.0	0.0	0.0	0.0
Puducherry							0.2	0.0	1.7	0.7	0.1	3.9

Table S8. The prevalence of mothers whose blood sample was not taken and 95% confidence interval for India and 36 states/union territories,1999 -2021

State	1999			2006			2016			2021		
	%	LCI	UCI	%	LCI	UCI	%	LCI	UCI	%	LCI	UCI
**India**	36.6	35.6	37.6	37.2	36.4	38.0	12.6	12.4	12.9	5.8	5.6	5.9
**States**												
Andhra Pradesh	20.7	16.4	25.9	10.7	8.6	13.2	1.3	0.9	1.9	0.4	0.2	0.9
Arunachal Pradesh	40.3	33.8	47.1	29.6	23.6	36.5	17.3	15.4	19.4	3.5	2.8	4.3
Assam	56.8	52.5	61.1	59.8	55.7	63.7	10.1	9.3	10.9	5.0	4.5	5.7
Bihar	44.7	40.1	49.5	33.2	28.4	38.5	36.6	35.4	37.8	19.7	18.8	20.6
Chhattisgarh	48.8	40.4	57.4	62.0	58.5	65.4	10.4	9.5	11.4	2.4	1.9	2.9
Goa	4.5	2.6	7.6	2.3	1.1	4.6	1.3	0.5	3.6	0.0	NA	NA
Gujarat	33.3	29.5	37.2	28.6	25.5	31.9	4.9	4.2	5.7	1.0	0.7	1.3
Haryana	19.4	16.1	23.3	39.2	33.4	45.4	5.0	4.3	5.8	1.7	1.3	2.1
Himachal Pradesh	24.4	20.9	28.2	20.6	16.8	25.0	2.5	1.7	3.8	0.8	0.4	1.6
Jharkhand	39.4	32.6	46.7	50.7	46.7	54.7	24.2	23.0	25.3	11.7	10.8	12.6
Karnataka	24.0	20.9	27.3	11.3	9.6	13.1	0.9	0.7	1.2	0.6	0.4	0.9
Kerala	3.8	2.4	6.0	1.4	0.7	3.2	0.4	0.2	1.1	0.6	0.2	1.4
Madhya Pradesh	52.3	49.1	55.4	51.3	48.0	54.5	10.0	9.4	10.7	3.1	2.7	3.5
Maharashtra	27.6	24.6	30.9	13.1	11.3	15.2	2.4	1.7	3.3	1.8	1.3	2.4
Manipur	65.3	60.8	69.6	33.0	30.3	35.9	6.6	5.8	7.5	2.0	1.5	2.8
Meghalaya	50.3	44.1	56.4	38.7	34.2	43.4	17.3	15.6	19.1	7.5	6.5	8.6
Mizoram	61.6	55.5	67.3	53.9	49.1	58.7	11.2	9.7	12.8	5.2	4.0	6.7
Nagaland	75.6	69.1	81.1	62.2	58.5	65.8	24.2	21.7	26.8	10.4	8.7	12.4
Odisha	47.5	43.9	51.1	49.5	45.7	53.3	5.0	4.5	5.7	1.7	1.4	2.2
Punjab	14.1	11.4	17.5	19.8	15.8	24.5	2.2	1.5	3.3	1.3	0.9	1.9
Rajasthan	43.1	39.9	46.4	49.8	46.4	53.2	10.2	9.5	10.9	4.0	3.6	4.5
Sikkim	54.8	48.9	60.6	25.3	21.1	29.9	1.6	0.8	2.9	2.5	1.3	4.8
Tamil Nadu	21.0	17.5	25.1	6.6	5.1	8.4	0.4	0.3	0.6	0.1	0.1	0.4
Telangana	16.8	12.9	21.6	10.7	8.6	13.2	1.2	0.7	1.8	0.4	0.2	0.7
Tripura	41.2	34.2	48.5	33.1	28.5	38.1	2.8	1.8	4.3	1.4	0.8	2.6
Uttar Pradesh	57.4	54.2	60.5	74.2	72.2	76.1	34.0	33.2	34.7	9.6	9.2	10.1
Uttarakhand	50.3	40.8	59.8	31.5	26.5	36.9	13.9	12.5	15.4	3.5	2.7	4.6
West Bengal	51.3	47.7	54.9	43.6	40.9	46.4	6.2	5.2	7.5	2.0	1.5	2.8
**Union territories**												
NCT Delhi	12.0	9.6	15.0	12.3	10.1	15.0	2.6	1.8	3.9	2.6	2.0	3.6
Jammu & Kashmir	14.0	11.7	16.7	12.0	9.7	14.7	2.9	2.4	3.5	1.0	0.8	1.4
Ladakh				12.0	9.7	14.7	2.1	1.1	3.9	1.0	0.4	2.5
Andaman & Nicobar							0.2	0.0	1.4	0.3	0.0	2.0
Chandigarh							4.6	1.7	11.7	0.0	NA	NA
D & N.H & D.D**							5.1	2.9	8.8	0.6	0.2	1.7
Lakshadweep							0.0	0.0	0.0	0.9	0.1	5.9
Puducherry							0.0	0.0	0.0	0.0	NA	NA

Table S9. The prevalence of mothers who have not taken a tetanus injection and 95% confidence interval for India and 36 states/union territories,1999 -2021

State	1999			2006			2016			2021		
	%	LCI	UCI	%	LCI	UCI	%	LCI	UCI	%	LCI	UCI
**India**	5.5	5.0	5.9	2.9	2.7	3.2	5.2	5.0	5.4	3.9	3.7	4.0
**States**												
Andhra Pradesh	6.5	4.1	10.1	3.3	2.2	4.8	2.2	1.7	3.0	4.7	3.7	5.8
Arunachal Pradesh	9.0	5.7	13.7	14.3	10.0	20.0	8.9	7.6	10.5	11.7	10.4	13.1
Assam	8.6	6.4	11.5	3.6	2.3	5.5	7.2	6.5	7.9	4.6	4.1	5.2
Bihar	11.1	8.5	14.5	7.0	4.7	10.5	3.2	2.7	3.7	4.1	3.6	4.6
Chhattisgarh	7.6	4.1	13.6	3.6	2.5	5.3	1.9	1.5	2.5	3.9	3.3	4.6
Goa	0.4	0.0	2.4	2.5	1.3	4.7	0.8	0.3	2.7	1.2	0.4	3.8
Gujarat	6.0	4.3	8.3	3.8	2.6	5.4	4.4	3.7	5.2	3.7	3.0	4.4
Haryana	1.3	0.6	2.9	2.4	1.1	5.2	3.4	2.8	4.1	2.9	2.5	3.5
Himachal Pradesh	0.7	0.2	1.9	4.9	3.1	7.6	4.6	3.5	5.9	5.2	3.6	7.4
Jharkhand	8.6	5.4	13.7	2.1	1.2	3.6	2.6	2.2	3.1	3.5	3.0	4.0
Karnataka	5.7	4.2	7.7	4.8	3.7	6.1	8.7	7.6	10.0	3.0	2.5	3.6
Kerala	5.7	4.0	8.2	1.2	0.5	2.9	1.7	1.0	2.8	1.9	1.2	3.1
Madhya Pradesh	6.3	5.0	8.0	2.7	1.8	4.0	3.1	2.8	3.5	3.0	2.7	3.5
Maharashtra	1.7	1.0	2.8	2.2	1.5	3.2	4.1	3.1	5.2	4.4	3.5	5.6
Manipur	5.6	3.8	8.1	2.3	1.5	3.4	3.8	3.1	4.6	3.3	2.5	4.4
Meghalaya	16.6	12.4	21.8	5.9	4.0	8.5	5.1	4.2	6.2	5.2	4.3	6.4
Mizoram	5.3	3.1	8.9	8.7	6.3	11.8	2.9	2.1	3.8	3.5	2.3	5.1
Nagaland	7.4	4.5	11.9	3.8	2.5	5.6	6.4	5.0	8.1	3.8	2.8	5.2
Odisha	3.4	2.3	4.9	4.5	3.1	6.4	4.4	3.9	5.1	2.0	1.6	2.4
Punjab	1.8	1.0	3.5	2.4	1.2	4.8	3.0	2.3	3.9	4.3	3.5	5.3
Rajasthan	8.4	6.7	10.4	3.7	2.6	5.2	3.3	2.9	3.7	3.0	2.6	3.4
Sikkim	5.9	3.7	9.4	1.1	0.4	3.0	1.4	0.7	2.7	5.9	3.4	10.1
Tamil Nadu	1.0	0.4	2.5	0.4	0.1	1.0	22.3	20.8	23.8	4.8	4.1	5.6
Telangana	2.8	1.4	5.5	3.3	2.2	4.8	4.8	3.6	6.4	7.2	6.4	8.2
Tripura	6.3	3.6	10.7	4.2	2.6	6.8	2.3	1.4	3.8	2.7	1.7	4.3
Uttar Pradesh	11.9	10.0	14.1	3.6	2.8	4.5	3.9	3.6	4.2	3.8	3.5	4.1
Uttarakhand	6.0	3.1	11.2	4.0	2.3	7.0	3.0	2.3	3.8	2.1	1.4	3.0
West Bengal	2.6	1.7	4.1	0.4	0.2	1.0	4.3	3.4	5.4	4.6	3.7	5.6
**Union territories**												
NCT Delhi	3.3	2.1	5.1	1.5	0.8	2.7	5.7	4.2	7.7	2.5	1.8	3.4
Jammu & Kashmir	4.5	3.2	6.2	3.7	2.5	5.5	6.6	5.8	7.5	7.4	6.6	8.4
Ladakh				3.7	2.5	5.5	5.4	3.7	7.9	6.5	4.5	9.4
Andaman & Nicobar							3.3	1.5	7.1	2.6	1.3	5.3
Chandigarh							4.9	1.8	12.3	0.0	NA	NA
D & N.H & D.D**							12.3	8.5	17.4	3.9	2.2	6.6
Lakshadweep							0.8	0.1	5.5	1.3	0.3	5.2
Puducherry							10.5	7.4	14.7	6.3	3.4	11.5

Table S10. The prevalence of mothers who have not taken iron and 95% confidence interval for India and 36 states/union territories,1999 -2021

State	1999			2006			2016			2021		
	%	LCI	UCI	%	LCI	UCI	%	LCI	UCI	%	LCI	UCI
**India**	19.6	18.8	20.4	18.6	17.9	19.3	16.7	16.4	17.0	11.0	10.7	11.2
**States**												
Andhra Pradesh	15.2	11.4	19.8	20.0	17.2	23.0	8.1	7.0	9.3	5.2	4.3	6.3
Arunachal Pradesh	13.8	9.8	19.0	19.5	14.5	25.7	18.1	16.2	20.1	11.9	10.7	13.3
Assam	13.3	10.6	16.6	18.0	15.0	21.4	9.7	9.0	10.5	4.8	4.3	5.4
Bihar	36.4	32.0	41.1	39.6	34.5	45.0	30.7	29.6	31.9	22.3	21.3	23.3
Chhattisgarh	24.5	17.9	32.6	15.9	13.4	18.8	6.7	6.0	7.5	6.1	5.3	7.1
Goa	3.6	2.0	6.6	12.0	9.0	15.9	4.2	2.4	7.3	0.3	0.0	2.0
Gujarat	12.3	9.9	15.3	10.0	8.0	12.3	17.4	15.9	18.9	9.9	9.0	11.0
Haryana	15.9	12.9	19.5	21.9	17.2	27.4	11.1	10.0	12.2	7.6	6.8	8.4
Himachal Pradesh	4.9	3.3	7.1	9.7	7.1	13.0	4.8	3.6	6.5	1.9	1.3	2.6
Jharkhand	28.8	22.7	35.8	28.9	25.4	32.7	25.1	23.9	26.3	13.8	12.9	14.8
Karnataka	10.9	8.8	13.4	18.2	16.2	20.4	12.4	11.2	13.6	10.5	9.6	11.6
Kerala	3.7	2.4	5.9	3.1	1.8	5.3	2.6	1.7	4.0	1.8	1.1	3.0
Madhya Pradesh	28.6	25.8	31.6	23.7	21.1	26.6	10.7	10.1	11.4	6.7	6.2	7.4
Maharashtra	8.1	6.4	10.2	13.6	11.8	15.6	14.9	13.3	16.5	13.6	11.8	15.7
Manipur	38.7	34.3	43.4	25.7	23.2	28.4	10.5	9.5	11.6	4.1	3.3	5.0
Meghalaya	10.0	6.8	14.4	18.7	15.4	22.6	9.7	8.4	11.1	7.3	6.3	8.5
Mizoram	21.6	17.0	27.1	28.8	24.6	33.4	10.9	9.2	12.8	9.5	7.4	12.0
Nagaland	31.6	25.6	38.4	57.6	53.9	61.3	32.6	29.8	35.5	17.4	15.0	19.9
Odisha	20.1	17.4	23.1	8.2	6.4	10.5	8.1	7.4	8.9	2.4	2.0	3.0
Punjab	12.5	9.9	15.7	21.9	17.8	26.7	9.1	7.8	10.5	9.0	7.8	10.3
Rajasthan	32.6	29.5	35.8	24.6	21.7	27.6	29.9	28.9	30.9	16.8	15.9	17.7
Sikkim	15.0	11.3	19.6	7.1	4.9	10.2	2.6	1.5	4.5	2.5	1.0	6.1
Tamil Nadu	6.9	4.9	9.7	7.0	5.6	8.9	5.2	4.4	6.1	1.2	0.9	1.8
Telangana	15.2	11.5	19.8	20.0	17.2	23.0	8.0	6.7	9.7	8.9	7.9	10.0
Tripura	14.6	10.2	20.5	16.5	13.1	20.7	11.1	9.0	13.7	8.2	6.2	10.6
Uttar Pradesh	37.8	34.7	40.9	25.8	23.9	27.8	29.6	28.8	30.3	13.7	13.2	14.2
Uttarakhand	31.9	23.6	41.4	11.5	8.3	15.6	14.9	13.4	16.5	9.2	7.7	10.9
West Bengal	21.2	18.4	24.4	12.6	10.9	14.6	8.7	7.3	10.2	3.3	2.6	4.2
**Union territories**												
NCT Delhi	11.4	9.0	14.2	13.2	10.8	16.0	6.9	5.1	9.2	4.8	3.9	5.9
Jammu & Kashmir	17.0	14.5	19.9	23.4	20.3	26.8	22.4	20.9	23.8	26.2	24.6	27.7
Ladakh				23.4	20.3	26.8	21.1	17.6	25.2	20.8	17.1	25.1
Andaman & Nicobar							7.2	4.4	11.6	4.1	2.2	7.6
Chandigarh							10.5	5.7	18.5	4.2	1.7	10.0
D & N.H & D.D**							15.2	11.0	20.8	11.9	8.7	15.9
Lakshadweep							5.7	2.7	11.4	1.6	0.4	6.3
Puducherry							5.2	3.2	8.3	1.0	0.4	2.3

Table S11. The prevalence of mothers who have not taken an ultrasound test and 95% confidence interval for India and 36 states/union territories,1999 -2021

State	1999			2006			2016			2021		
	%	LCI	UCI	%	LCI	UCI	%	LCI	UCI	%	LCI	UCI
**India**	78.9	78.1	79.7	64.7	63.9	65.5	23.3	23.0	23.6	12.7	12.5	13.0
**States**												
Andhra Pradesh	77.0	71.7	81.5	59.0	55.3	62.5	3.1	2.4	3.9	2.8	2.1	3.8
Arunachal Pradesh	94.4	90.3	96.8	34.1	27.8	41.1	36.7	34.2	39.3	13.1	11.8	14.6
Assam	96.2	94.4	97.4	14.1	11.6	17.0	42.9	41.5	44.2	16.7	15.7	17.7
Bihar	92.0	89.0	94.2	25.3	21.1	30.1	51.0	49.8	52.3	29.8	28.8	30.9
Chhattisgarh	90.1	83.7	94.2	12.3	10.2	14.6	40.4	38.9	42.0	21.5	20.2	22.9
Goa	36.9	31.5	42.7	86.5	82.5	89.6	3.3	1.6	6.6	1.9	0.9	4.1
Gujarat	62.8	58.8	66.7	45.7	42.2	49.2	13.5	12.3	14.8	6.7	6.0	7.5
Haryana	79.6	75.6	83.0	53.1	46.9	59.2	6.3	5.5	7.2	3.6	3.1	4.2
Himachal Pradesh	83.6	80.4	86.4	52.5	47.6	57.4	5.7	4.4	7.5	3.6	2.7	4.9
Jharkhand	93.8	89.2	96.5	15.9	13.4	18.8	48.1	46.7	49.4	28.6	27.4	29.8
Karnataka	73.7	70.2	76.8	59.1	56.4	61.8	4.0	3.2	4.9	3.7	3.1	4.4
Kerala	54.9	50.5	59.3	77.7	73.4	81.4	0.6	0.3	1.4	2.4	1.7	3.6
Madhya Pradesh	88.1	85.8	90.0	18.0	15.7	20.6	38.8	37.7	39.9	24.4	23.4	25.4
Maharashtra	65.9	62.8	69.0	57.1	54.4	59.8	11.0	9.8	12.3	4.2	3.4	5.1
Manipur	86.8	83.4	89.7	28.0	25.4	30.6	16.0	14.8	17.2	6.3	5.5	7.3
Meghalaya	93.7	89.9	96.1	25.4	21.7	29.5	58.2	56.0	60.3	43.8	41.6	46.1
Mizoram	91.9	88.0	94.6	15.5	12.3	19.4	34.7	32.1	37.4	21.2	18.6	24.1
Nagaland	96.7	92.8	98.5	17.8	15.2	20.7	37.1	34.3	40.0	28.9	26.3	31.6
Odisha	95.7	94.1	96.9	19.2	16.4	22.2	28.6	27.4	29.8	14.3	13.3	15.3
Punjab	76.8	73.0	80.2	61.4	56.0	66.5	3.5	2.6	4.8	3.4	2.8	4.2
Rajasthan	81.9	79.2	84.4	26.1	23.3	29.1	17.9	17.1	18.8	5.7	5.2	6.3
Sikkim	87.8	83.2	91.2	44.3	39.6	49.2	4.9	3.4	7.0	3.7	1.6	8.3
Tamil Nadu	60.8	56.3	65.1	56.1	52.8	59.3	3.6	3.0	4.4	0.9	0.6	1.3
Telangana	67.3	61.7	72.4	59.0	55.3	62.5	3.0	2.2	4.1	2.6	2.1	3.2
Tripura	91.0	85.9	94.5	21.8	18.0	26.2	41.4	37.6	45.3	19.2	16.4	22.4
Uttar Pradesh	86.0	83.6	88.1	16.8	15.2	18.5	35.2	34.4	36.0	14.2	13.7	14.8
Uttarakhand	73.3	63.6	81.2	41.1	35.7	46.7	13.1	11.8	14.6	6.1	5.0	7.5
West Bengal	92.4	90.6	93.9	19.0	17.0	21.1	30.2	28.0	32.5	10.8	9.6	12.2
**Union territories**												
NCT Delhi	58.3	54.2	62.3	63.8	60.0	67.5	4.0	2.8	5.6	3.4	2.6	4.4
Jammu & Kashmir	87.2	84.8	89.3	43.1	39.4	47.0	5.8	5.1	6.5	2.2	1.8	2.7
Ladakh				43.1	39.4	47.0	3.9	2.5	6.0	2.3	1.3	4.3
Andaman & Nicobar							4.0	2.9	5.6	3.0	1.6	5.6
Chandigarh							3.3	1.1	10.0	4.3	1.8	9.9
D & N.H & D.D**							10.0	6.5	15.1	8.4	5.9	11.8
Lakshadweep							0.9	0.1	6.4	6.4	3.3	12.4
Puducherry							4.9	2.8	8.5	0.1	0.0	0.2

Table S12. Summary distribution of inadequate antenatal care prevalence across states/union territories of India, 1999-2021

Year	1999 (n=30)	2006 (n=31)	2016 (n=36)	2021 (n=36)
Mean	86.2	72.6	35.3	23.1
Std. dev.	13.3	19.4	19.8	13.3
Variance	176.3	376.8	392.5	177.1
1%	44.4	26.5	5.9	4.3
25%	79.9	55.9	18.9	13.3
50%	92.8	81.4	32.2	20.3
75%	95.5	87.6	51.0	32.1
99%	99.0	94.1	74.2	55.0

Table S13. State-level Spearman's rank correlation (p-value) in the prevalence of "having received inadequate quality of ANC" across four waves (1999-2021)

Year	1999 (n=15,061)	2006(n=23,137)	2016(n=127,515)	2021(n=131,842)
**1999**	1			
**2006**	0.890 (p<0.001)	1		
**2016**	0.757 (p<0.001)	0.857 (p<0.001)	1	
**2021**	0.731 (p<0.001)	0.846 (p<0.001)	0.894 (p<0.001)	1

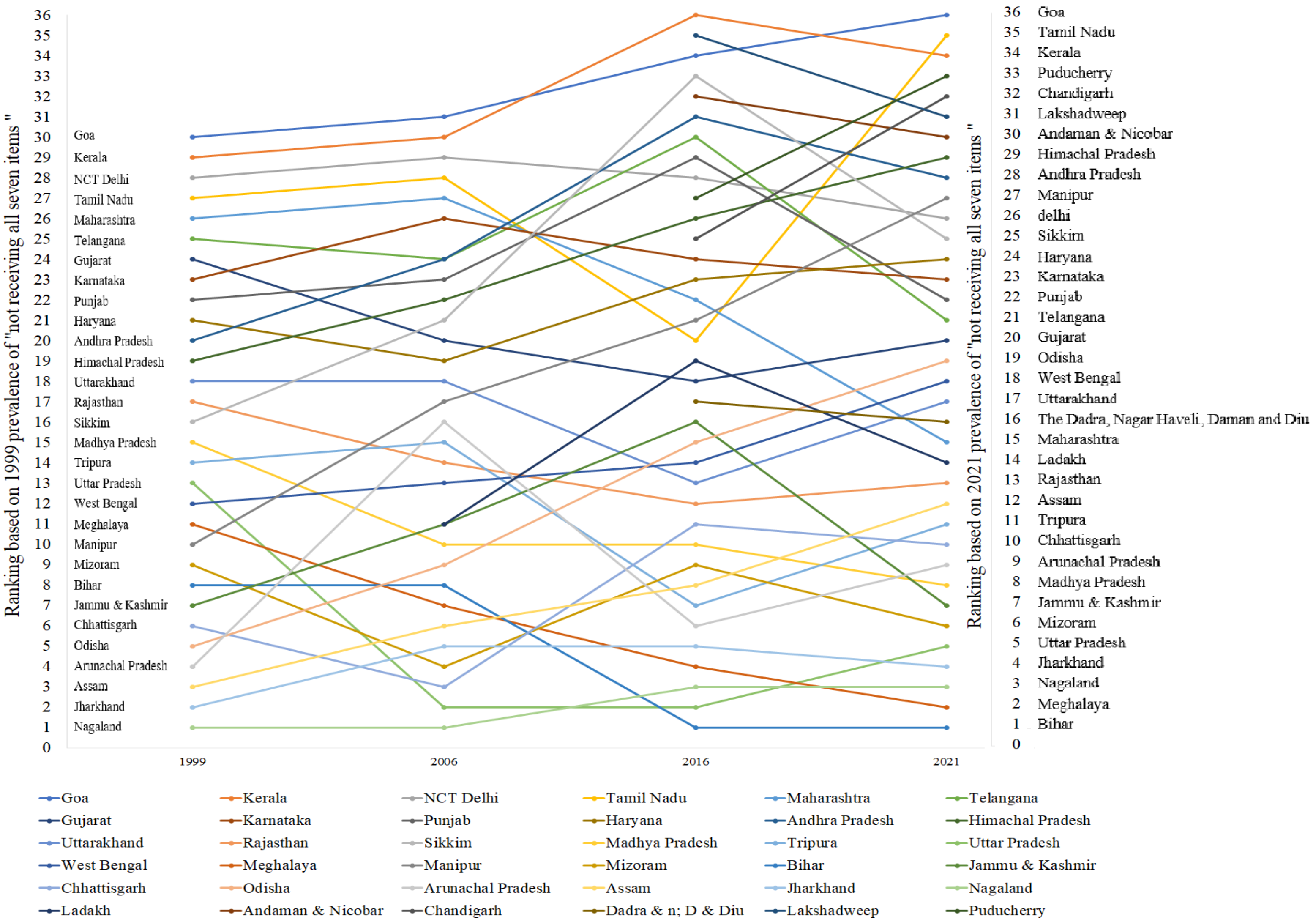

Figure S1.Ranked ordering of weighted prevalence of mothers who have received inadequate quality of ANC by states and union territories of India in1999, 2006, 2016 and 2021

Figure S2. Gap in the prevalence of receiving individual ANC services between the highest and lowest socioeconomic status

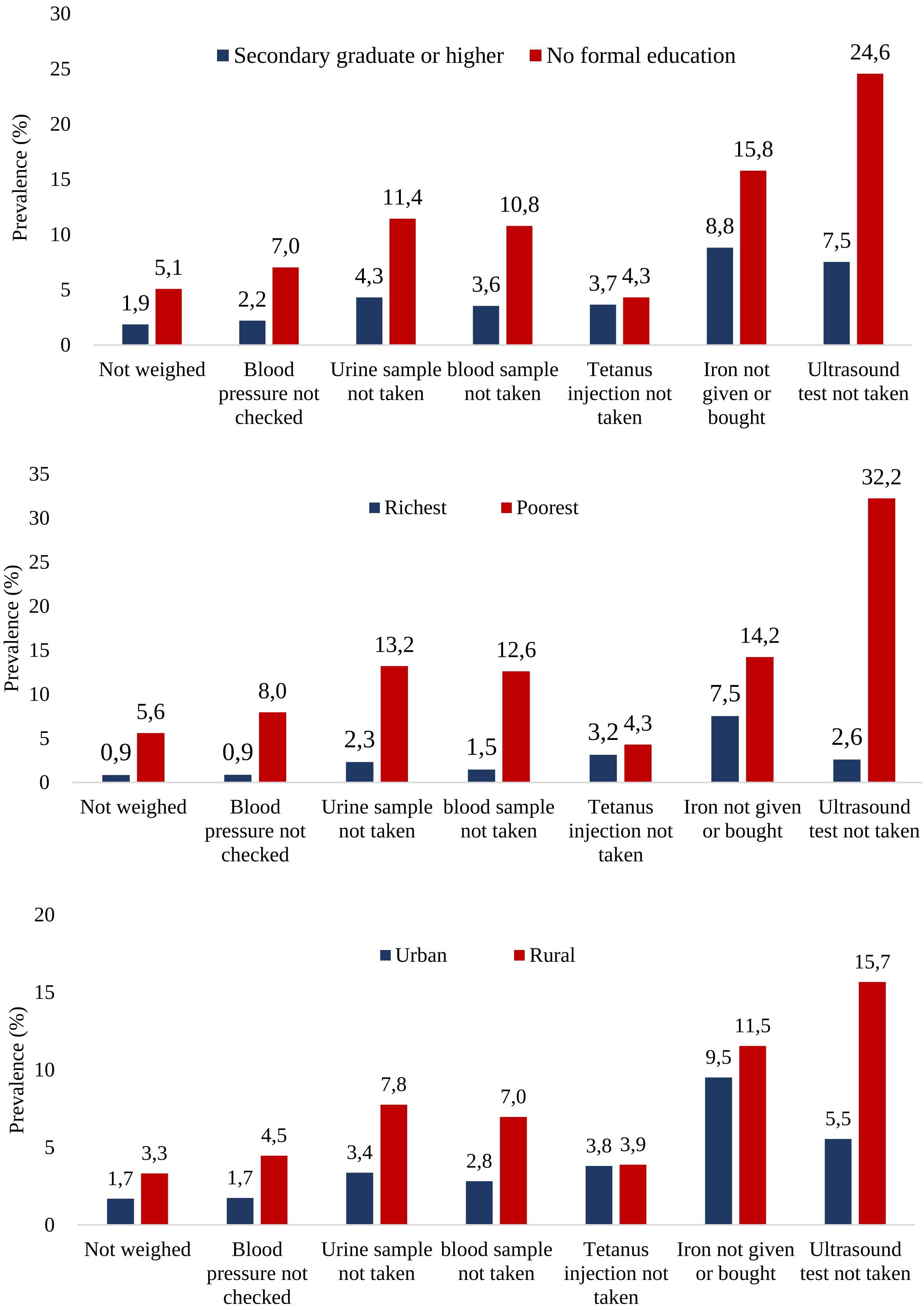

Supplement: Online Supplementary Document [file jogh-14-04188-s001.pdf]
